# Combined Inhibition of p97 and the Proteasome Causes Lethal Disruption of the Secretory Apparatus in Multiple Myeloma Cells

**DOI:** 10.1371/journal.pone.0074415

**Published:** 2013-09-17

**Authors:** Holger W. Auner, Anne Marie Moody, Theresa H. Ward, Marianne Kraus, Enrico Milan, Philippa May, Aristeidis Chaidos, Christoph Driessen, Simone Cenci, Francesco Dazzi, Amin Rahemtulla, Jane F. Apperley, Anastasios Karadimitris, Niall Dillon

**Affiliations:** 1 Gene Regulation and Chromatin Group, MRC Clinical Sciences Centre, Imperial College London, London, United Kingdom; 2 Centre for Haematology, Department of Medicine, Imperial College London, London, United Kingdom; 3 Immunology and Infection Department, London School of Hygiene and Tropical Medicine, London, United Kingdom; 4 Kantonsspital, St. Gallen, St. Gallen, Switzerland; 5 Age Related Diseases Group, Division of Genetics and Cell Biology, San Raffaele Scientific Institute, Milano, Italy; Johns Hopkins School of Medicine, United States of America

## Abstract

Inhibition of the proteasome is a widely used strategy for treating multiple myeloma that takes advantage of the heavy secretory load that multiple myeloma cells (MMCs) have to deal with. Resistance of MMCs to proteasome inhibition has been linked to incomplete disruption of proteasomal endoplasmic-reticulum (ER)-associated degradation (ERAD) and activation of non-proteasomal protein degradation pathways. The ATPase p97 (VCP/Cdc48) has key roles in mediating both ERAD and non-proteasomal protein degradation and can be targeted pharmacologically by small molecule inhibition. In this study, we compared the effects of p97 inhibition with Eeyarestatin 1 and DBeQ on the secretory apparatus of MMCs with the effects induced by the proteasome inhibitor bortezomib, and the effects caused by combined inhibition of p97 and the proteasome. We found that p97 inhibition elicits cellular responses that are different from those induced by proteasome inhibition, and that the responses differ considerably between MMC lines. Moreover, we found that dual inhibition of both p97 and the proteasome terminally disrupts ER configuration and intracellular protein metabolism in MMCs. Dual inhibition of p97 and the proteasome induced high levels of apoptosis in all of the MMC lines that we analysed, including bortezomib-adapted AMO-1 cells, and was also effective in killing primary MMCs. Only minor toxicity was observed in untransformed and non-secretory cells. Our observations highlight non-redundant roles of p97 and the proteasome in maintaining secretory homeostasis in MMCs and provide a preclinical conceptual framework for dual targeting of p97 and the proteasome as a potential new therapeutic strategy in multiple myeloma.

## Introduction

Multiple myeloma (MM) is a tumour of transformed plasma cells, terminally differentiated B cells that are highly specialised to synthesise and secrete large amounts of immunoglobulins (Ig) [1]. The production of large quantities of secreted protein places a substantial strain on the protein-folding machinery and can result in the accumulation of misfolded/unfolded proteins, triggering the unfolded protein response (UPR). The UPR is a fundamental cellular process that acts to maintain secretory homeostasis by attenuating overall protein translation, expanding the endoplasmic reticulum (ER), and increasing ER-associated protein degradation (ERAD) of misfolded proteins in the cytosol by the proteasome [2-4]. Failure of the UPR results in overwhelming ER stress and apoptosis [4].

Plasma cells and MM cells (MMCs) have a well-developed secretory apparatus to accommodate Ig secretion but are nevertheless highly sensitive to agents that interfere with intracellular protein metabolism [5-7]. This forms the basis for selective targeting of MMCs by the proteasome inhibitor bortezomib, which is widely used for the treatment of MM [8]. The combined findings of several studies suggest that the preferential toxicity of proteasome inhibitors to MMCs can be attributed, to a large extent, to ERAD impairment and overwhelming ER stress [9-12]. Primary or acquired resistance of MMCs to bortezomib is an important clinical problem and has been linked to incomplete ERAD disruption and the activation of alternative protein degradation pathways, such as autophagy and aggresome formation [5,13-15]. This makes it important to develop therapeutic approaches that give more effective disruption of secretory homeostasis in MMCs [1].

Prior to proteasomal degradation, misfolded proteins are exported from the ER lumen to the cytosolic side of the ER, where they are earmarked for degradation by poly-ubiquitination [16]. This acts as a signal for binding of the cytosolic ATPase, p97 (also known as valosin-containing protein, VCP; or Cdc48), which delivers the ubiquitinated protein to the proteasome by translating ATP hydrolysis into mechanical force [17-21]. Hence, p97 has a central role in ERAD and this role is closely linked to the proteasome. However, like the proteasome, p97 also mediates the degradation of non-secretory proteins that regulate a variety of cellular functions [22-25]. Inhibitors of p97, were recently reported to kill cancer cells by mechanisms that are related to disruption of the secretory apparatus [26-30]. Eeyarestatin 1 (Eer1), which binds to both p97 and the ER membrane, preferentially induces cancer cell death by ERAD disruption and effects that are similar to those induced by bortezomib, including ER stress-induced transcriptional up-regulation of NOXA and induction of CHOP [26,28]. *N* ^*2*^ *,N* ^*4*^-dibenzylquinazoline-2,4-diamine (DBeQ), a reversible inhibitor of the p97 ATPase activity, was found to be a potent activator of caspases in cancer cells and to inhibit both ERAD and autophagosome maturation [26,27]. Furthermore, there is evidence that sorafenib, a multikinase inhibitor of the Raf/MEK/ERK pathway and of tyrosine kinase receptors, induces cancer cell death by preventing p97 tyrosine phosphorylation and consecutive disruption of secretory homeostasis [30,31]. Thus, p97 inhibition has the potential to overcome some limitations of proteasome inhibition as an anti-cancer strategy, particularly in secretory cancers such as MM [32,33].

The essential roles of p97 and the proteasome in regulating protein degradation, which are closely linked in ERAD but also show a considerable degree of non-redundancy, open up the possibility that simultaneous inhibition of p97 and the proteasome could be highly efficient in disrupting secretory homeostasis in MMCs. Here we demonstrate that inhibitors of either p97 or the proteasome have quite different effects on the secretory apparatus in MMCs, and that these effects vary substantially between different MMC lines, and between MMCs and non-secretory or non-transformed cells. Moreover, we show that dual inhibition of p97 and the proteasome has dramatic effects on ER morphology and protein turnover that are selective for MMCs and are very different from the effects of single inhibition with either drug. Our results provide a conceptual preclinical framework for simultaneous targeting of p97 and the proteasome as a potential new therapeutic strategy in multiple myeloma.

## Materials and Methods

### Ethics statement

Bone marrow myeloma cells, bone marrow mesenchymal stroma cells, and skin fibroblasts were collected at the Imperial College London Centre for Haematology after approval by the National Research Ethics Service Committee East of England – Cambridge Central and obtaining signed informed consent in accordance with the Declaration of Helsinki.

### Cell culture and reagents

The human MMC lines OPM-2, RPMI 8226, U-266, KMS-11, and parental/bortezomib-adapted AMO-1 have been described previously [7,15]. Primary MMCs were isolated from bone marrow aspirates using CD138 microbeads and MACS columns (Miltenyi) and cultured as MMC lines with the addition of 10ng/ml human recombinant interleukin (IL)-6 (Peprotech). Primary human skin fibroblasts and peripheral blood mononuclear cells were cultured in the same medium but without IL-6. Eer1 (Tocris), DBeQ (Cambridge Bioscience), zVADfmk (Calbiochem), SP600125 (Sigma), and bortezomib (Velcade®, Millenium) were stored in single-use aliquots at -20°C.

### Analysis of apoptosis

Apoptosis was assessed by staining with Annexin V-FITC and propidium iodide (PI; BD Pharmingen) as previously described [7]. Cells negative for Annexin V-FITC and PI were classified as live.

### Immunoblotting

Whole cell lysates were prepared as previously described [7]. Primary antibodies were directed against Akt, phospho-Akt (Ser473), caspase-3, p58^IPK^, GRP94, eIF2α, phospho-eIF2α (Ser51), S6 ribosomal protein, phospho-S6 ribosomal protein (Ser235/236), β-tubulin (all from Cell Signaling Technologies), and ubiquitin (P4D1, Santa Cruz Biotechnology).

### Qualitative and quantitative ER staining

Cells were incubated with 100nM BODIPY FL brefeldin A (BFA-BODIPY, Invitrogen) at 37°C for 40 minutes in the dark, washed and resuspended in phosphate-buffered saline (PBS), and analysed immediatedy on a BD LSR II flow cytometer. For morphological ER analysis, cells were incubated in medium with 1µM ER Tracker Blue-White DPX (Molecular Probes) at 37°C for 15 minutes, pelleted, and resuspended in 50µl medium. The cell suspension was applied to an imaging chamber [34], and imaged with a Zeiss LSM510 or Leica SP5 confocal microscope.

### Electron microscopy

1x10^7^ cells were fixed, washed, post-fixed and embedded as previously described [35]. Ultrathin sections were cut on a Leica Ultracut R microtome, stained with Reynolds lead citrate, and observed in a Jeol JEM 1200EX II transmission electron microscope.

## Results

### Dual inhibition of p97 and the proteasome is highly toxic to myeloma cells

**Figure 1:**
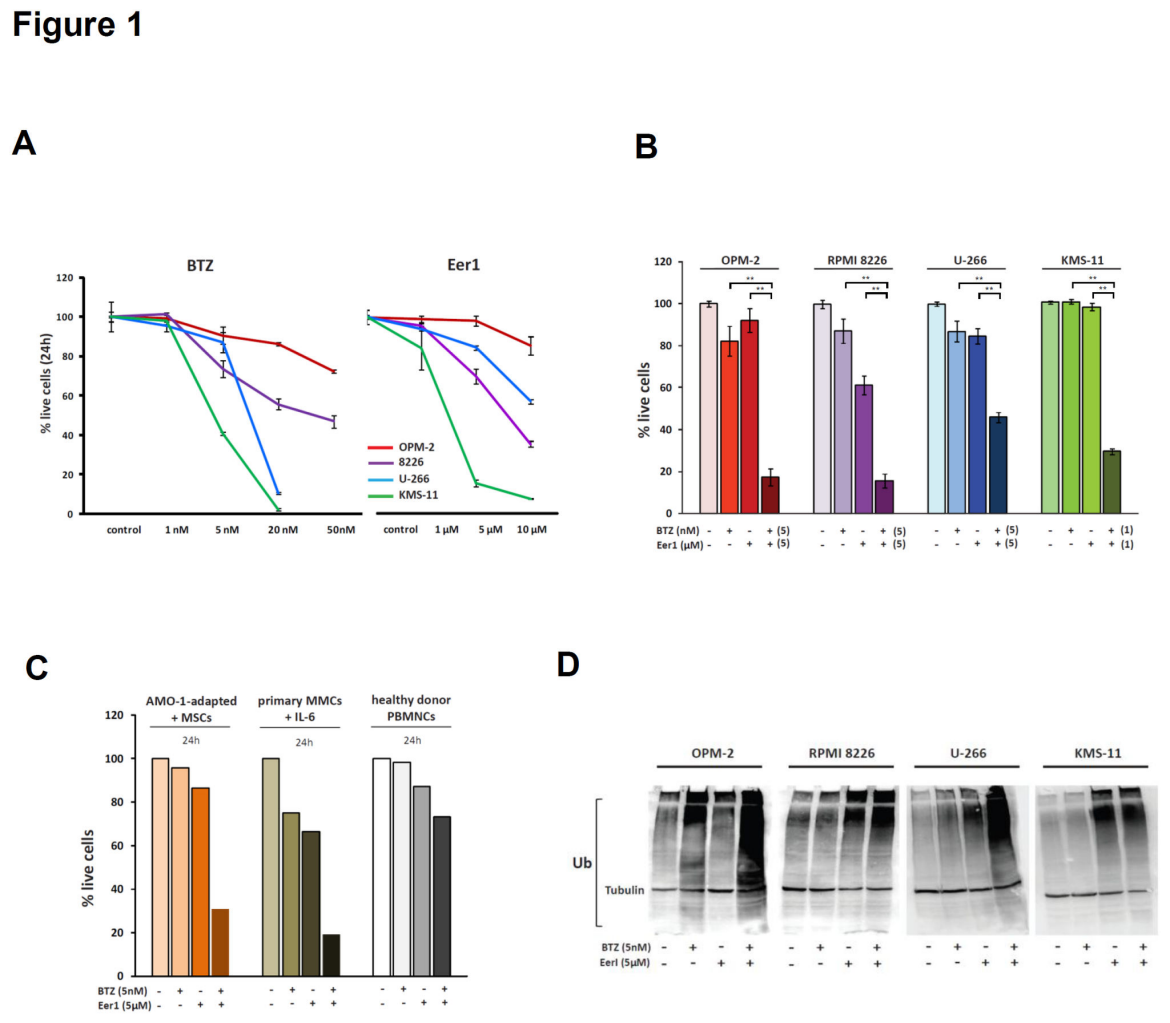
Dual p97 and proteasome inhibition induces high levels of apoptosis and disrupts protein degradation in MMCs. (**A**) A panel of human MMC lines were treated with the indicated concentrations of bortezomib (BTZ) or Eer1 for the indicated time. The proportion of live cells relative to DMSO-treated controls was determined by staining with Annexin V-FITC and PI (mean and SEM of 3 independent experiments). (B) MMC lines were treated with the indicated concentrations of BTZ and Eer1 for 48h (OPM-2, RPMI 8226) or 24h (U-266, KMS-11) and the proportion of live cells compared to controls determined as in A (*p<.05, **p<.001, two-sided student’s t-test). (**C**) BTZ-adapted AMO-1 MMCs co-cultured with human bone marrow MSCs, primary MMCs grown in the presence of IL-6, and healthy donor PBMNCs were subjected to single and dual inhibition with Bortezomib and Eer1 (the median of 3 technical replicates is shown). (**D**) Immunoblotting for ubiquitinated proteins and tubulin (loading control) carried out on whole cell extracts prepared from MMC lines treated with bortezomib, Eer1, or both inhibitors, for 24h (14h in KMS-11 cells due to their higher apoptotic sensitivity).

We first determined the apoptotic sensitivity of a panel of human MMC lines to bortezomib and the p97 inhibitor Eer1 (Figure 1A). The result of this analysis shows that OPM-2 cells were the most resistant to both bortezomib and Eer1, while KMS-11 cells were the most sensitive to both inhibitors. Next, we investigated whether simultaneous inhibition of p97 and of the proteasome increases MMC death compared to separate inhibition. We treated the panel of MMC lines with concentrations of bortezomib (5nM) and Eer1 (5µM) that have been shown to be pharmacologically effective in cancer cells, including malignant B cells, and fibroblasts [11,29,36-38]. . In addition, we found that a 2-hour treatment with 5nM of bortezomib blocked chymotrypsin-like proteasome activity by approximately 80% in OPM-2 cells, and that an 8-hour treatment of KMS-11 cells with Eer1, but not with bortezomib, significantly up-regulated HMOX1 levels, as demonstrated previously (data not shown) [11]. At these concentrations, and at the time-points studied, bortezomib and Eer1 had little impact on MMC line survival on their own (Figure 1B). However, dual p97/proteasome inhibition induced apoptosis very effectively in all MMC lines investigated (Figure 1B). Importantly, OPM-2 cells, which are resistant to bortezomib and characterised by abnormalities that confer poor outcome in patients, including t(4;14) and constitutive Akt activation, were also killed effectively by dual p97/proteasome inhibition [39,40]. To extend these findings, we treated the bortezomib-adapted MMC line AMO-1a, which we cultured in the presence of primary human bone marrow stroma cells to confer further pro-survival signals, with bortezomib and Eer1. We also tested the effect of separate and dual p97/proteasome inhibition on patient-derived MMCs grown in the presence of IL-6, and on healthy donor peripheral blood mononuclear cells (PBMNCs). While dual p97/proteasome inhibition effectively killed both the AMO-1a cell line and the primary MMCs, it was substantially less toxic against PBMNCs (Figure 1C). These findings demonstrate that combined p97/proteasome inhibition is highly toxic to MMCs, including MMC lines with primary and secondary resistance to bortezomib.

Increased intracellular levels of ubiquitinated proteins are considered to indicate impaired protein degradation by the proteasomal machinery [5,15,41]. To test how separate and combined inhibition of p97 and the proteasome affects protein degradation, we quantified the levels of ubiquitinated proteins in whole cell extracts from four MMC lines (Figure 1D). Dual inhibition of p97 and the proteasome led to increased levels of ubiquitinated proteins in all MMC lines tested, indicating effective disruption of gross protein degradation. However, an increased effect of dual over separate inhibition on levels of ubiquitinated proteins was only observed in two lines (OPM-2 and U-266). It is also worth noting that bortezomib induced an increase in ubiquitinated proteins only in OPM-2 cells, while inhibition of p97 with Eer1 led to increased levels of ubiquitinated proteins in the RPMI8226, U-266, and KMS-11 cells. Of particular importance is the finding that increased levels of ubiquitinated proteins after separate inhibition did not generally correlate with cell death (Figure 1B). This observation strongly suggests that additional mechanisms are involved in the induction of cell death by inhibitors of ERAD pathways.

### Combined inhibition of p97 and the proteasome drastically disrupts ER configuration in MMCs

**Figure 2:**
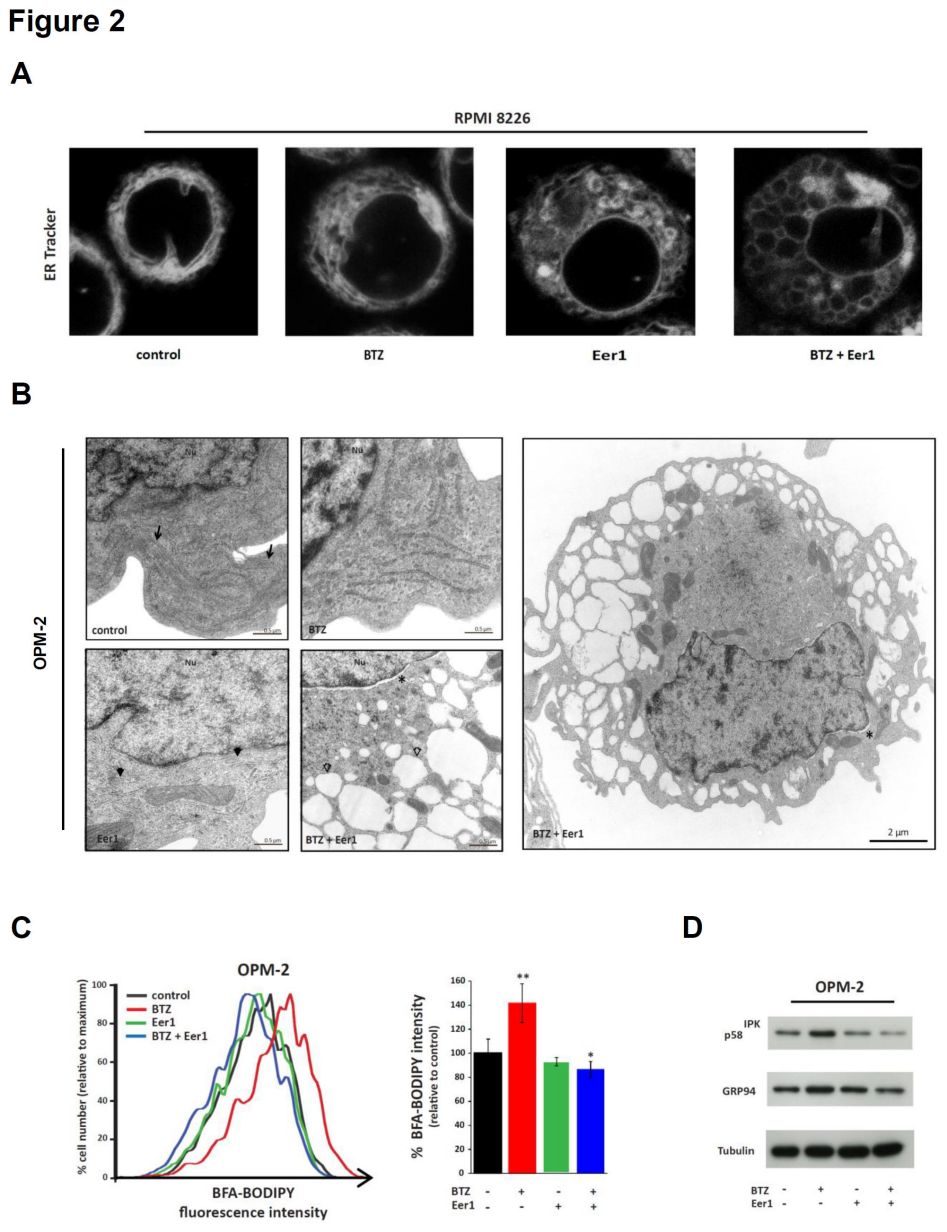
Combined inhibition of p97 and the proteasome dramatically affects ER configuration. (**A**) RPMI 8226 myeloma cells were stained with ER Tracker Blue-White DPX following treatment for 24h with bortezomib (BTZ; 5nM), Eer1 (5µM), or both. Representative confocal microscopic images show minor ER alterations after BTZ treatment, transformation of tubule-lamellar into globular ER structures after treatment with Eer1, and widespread ER vacuolisation after dual treatment. (**B**) Representative electron microscopic images of OPM-2 cells after treatment with BTZ (5nM), Eer1 (5µM), or BTZ and Eer1, for 24h. Arrows indicate classical ER in control cells, black arrowheads indicate moderately dilated and disrupted ER in Eer1-treated cells, and open arrowheads indicate vacuolised ER with reduced ribosomes on the cytosolic ER surface. Another cell treated with Eer1 and BTZ is shown at lower magnification (right panel). Areas of dilated perinuclear space are indicated by asterisks. Nu, nucleus. (**C**) BTZ and Eer1 have different effects on ER volume as shown by staining of OPM-2 cells with BFA-BODIPY after treatment for 24h with BTZ (5nM), Eer1 (5µM), or with BTZ and Eer1. A representative histogram (left panel) and the mean and SEM of 6 experiments (right panel) are shown (*p<.05, **p<.001, two-sided student’s t-test). (**D**) Immunoblotting for lumenal ER chaperones and tubulin (loading control) was carried out on whole cell extracts prepared from OPM-2 cells treated as in (C).

Inhibitors of the proteasome and of p97 cause ER stress in MM and other tumour cells, and ER stress is known to induce changes in ER structure [9-11,26,42-44]. To investigate the effects of separate and combined inhibition of p97 and the proteasome on ER structure in MMCs, we visualised the ER of MMC lines after staining with the fluorescent ER dye ER Tracker Blue-White DPX (Figure 2A and Figure S1). In most of the control cells, the ER was visible as elongated tubular structures with some small globular regions. Bortezomib did not cause any substantial changes in ER structure, although the ER appeared to be slightly more densely packed in some cells. However, treatment with the p97 inhibitor Eer1 had a clear effect on the ER. Most of the tubular ER was lost, and small to medium-sized vesicular and globular structures were instead observed. Dual inhibition with bortezomib and Eer1 caused a different and dramatic effect in that large parts of the ER were transformed into medium-sized to large vacuoles. This vacuolisation of the ER was not observed after treatment with high doses of bortezomib, Eer1, or tunicamycin (Figure S2), indicating that the changes were not caused by overwhelming ER stress generated by an additive effect of the two inhibitors. Furthermore, pre-incubation with the pan-caspase inhibitor zVADfmk did not abrogate ER vacuolisation, indicating that it did not occur as an unspecific consequence of apoptotic signalling (Figure S2).

To further investigate the changes in ER configuration we used transmission electron microscopy (TEM) of OPM-2 cells (Figure 2B and Figure S3), which showed results that were consistent with our findings obtained by staining with ER Tracker Blue-White DPX. The TEM analysis of untreated cells showed the expected elongated and narrow tubular ER structures with ribosomes attached to the cytosolic ER surface. Bortezomib did not have a uniformly discernible impact on ER structure, although the ER appeared slightly distended and less elongated in some cells. Inhibition of p97 with Eer1 did have an impact on ER morphology at the level of ultrastructure, giving rise to relatively short and moderately dilated tubular structures in most cells. Dual inhibition had a much more dramatic effect with the ER appearing predominantly as very large vacuoles in the majority of cells. Dual p97/proteasome inhibition also resulted in an empty appearance of the ER lumen, and ribosomes were largely absent from the cytosolic surface of the ER. The observation that the perinuclear space was also substantially dilated, had the same empty appearance and showed loss of ribosomes from its surface as the vacuoles, provides strong support for the notion that the vacuoles are indeed derived from the ER. The finding that most cells presenting with vacuolised ER did not show any apoptotic chromatin condensation further indicates that the changes in ER morphology are unlikely to be unspecific consequences of apoptotic signalling.

We next investigated how dual p97/proteasome inhibition affects cellular ER content by staining OPM-2 cells with the fluorescent brefeldin A derivative, BFA-BODIPY (Figure 2C), and immunoblotting for key ER luminal chaperones (Figure 2D) [45]. While bortezomib treatment increased the amount of ER stained by BFA-BODIPY by approximately 40% in OPM-2 cells, Eer1 on its own had no discernible impact. Dual p97/proteasome inhibition not only blocked the increase in BFA-BODIPY staining that was induced by bortezomib, but reduced it compared to control cells. Consistent with these findings, immunoblotting showed an increase in the levels of the ER lumenal chaperones, p58^IPK^ (DNAJC3) and GRP94, after proteasome inhibition, and a decrease after dual ERAD inhibition, while Eer1 had no detectable effect on the levels of either protein. Together, these observations indicate that bortezomib increases ER content in MMCs, whereas inhibition of p97 with Eer1 causes moderate alterations to ER structure. In contrast, dual p97/proteasome inhibition drastically alters the configuration of the ER.

### Dual inhibition of p97 and the proteasome disrupts key pathways that control survival, death, and protein synthesis and induces predominantly caspase-dependent cell death in MMCs

**Figure 3:**
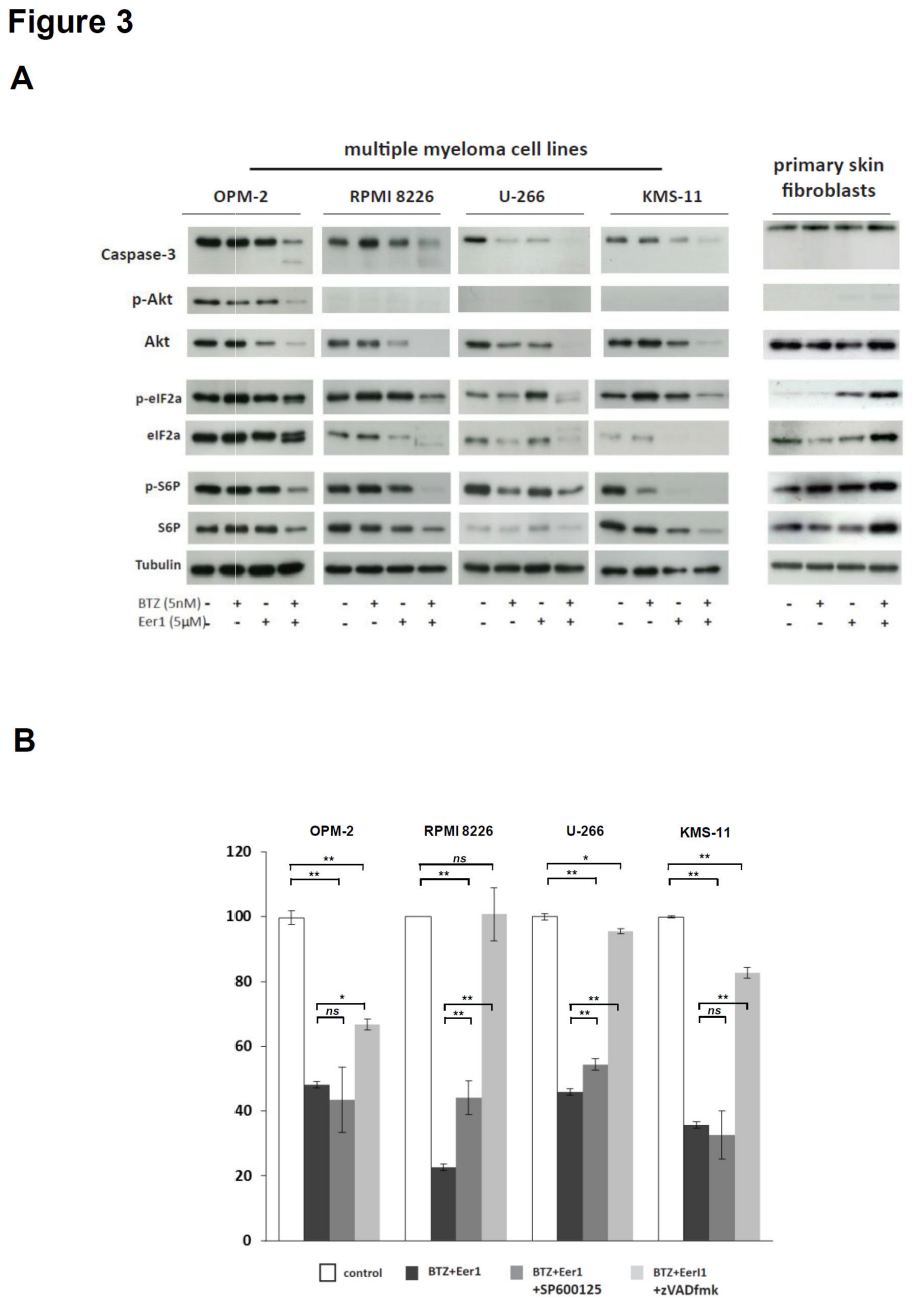
Dual p97/proteasome inhibition deregulates key cell survival and protein translation control pathways in MMCs. (**A**) Immunoblotting for the indicated survival- and apoptosis-related proteins was carried out on whole cell extracts from MMC lines and primary human fibroblasts prepared after treatment with either BTZ, Eer1, or both inhibitors, for 24h (14h in KMS-11 cells due to their higher apoptotic sensitivity to inhibitors). (**B**) Cell death induced by dual p97/proteasome inhibition is predominantly caspase-dependent and JNK-independent. The proportion of live cells was determined after treatment with BTZ (5nM) and Eer1 (5µM) with or without the pan-caspase inhibitor zVADfmk (50µM) or the JNK inhibitor SP600125 (10µM) for 24h (36h for OPM-2 cells; all values are the mean and SEM of 3 experiments; *p<.05, **p<.001, two-sided student’s t-test).

Next, we investigated the impact of single (bortezomib or Eer1 alone) and dual p97/proteasome inhibition on key signalling pathways in human MMC lines, and compared the effects with those induced in untransformed primary human fibroblasts (Figure 3A). Consistent with the induction of MMC death (Figure 1B), dual inhibition resulted in activation of the critical apoptotic executioner, caspase-3, as indicated by proteolytic processing of full-length caspase-3, in all four MMC lines investigated. This effect was observed to a much lesser extent after treatment with bortezomib or Eer1 alone. Importantly, there was no evidence that dual p97/proteasome inhibition activates caspase-3 in fibroblasts.

Activation of the Akt pathway, as indicated by phosphorylation of Akt, is an important survival signal that has been shown to occur in a proportion of MMCs [40]. In OPM-2 cells, which have constitutively active Akt (indicated by phosphorylation) [46], treatment with bortezomib or Eer1 caused a mild decrease in Akt phosphorylation, while dual inhibition abolished Akt phosphorylation. While phospho-Akt was not detectable in the other MMC cell lines tested here, dual inhibition also caused a substantial decrease in overall Akt protein levels, whereas treatment with bortezomib or Eer1 alone had no or a moderate effect. Importantly, single or dual inhibition had no effect on Akt levels in fibroblasts, providing further evidence of the selective effects of dual p97/proteasome inhibition on MMCs.

The observed changes in ER configuration (Figure 2) and Akt signalling that we observed in MMCs prompted us to investigate the impact of bortezomib and Eer1 on selected protein translation control pathways in these cells. Phosphorylation of the α-subunit of the eukaryotic translation initiation factor 2 (eIF2α) on Ser51 results in attenuation of protein translation and indicates activation of a PERK-dependent UPR signalling that results in attenuated protein translation [2,47]. We observed a moderate increase in eIF2α phosphorylation in response to bortezomib in three of the four MMC lines (OPM-2, RPMI-8226, KMS-11) investigated and following treatment with Eer1 in two lines (RPMI-8226, U-266). In contrast, dual inhibition invariably caused proteolytic processing and/or loss of phosphorylated and total eIF2α, which has been linked to terminal translational shut-down rather than adaptive attenuation of translation [48-50]. In fibroblasts, Eer1 alone and particularly dual inhibition with Eer1 and bortezomib induced phosphorylation of eIF2α on Ser51, indicating activation of adaptive PERK signalling. Phosphorylation of ribosomal protein S6 (S6P) correlates with an increase in translation of mRNA transcripts that contain an oligopyrimidine tract in their 5' untranslated regions [51]. Dual p97/proteasome inhibition substantially decreased levels of phosphorylated and total S6P in all MMCs lines, whereas treatment with only bortezomib or Eer1 resulted in a reduction in the levels of phosphorylated and total S6P in two of the four MMC lines in each case (U-266 and KMS-11 for bortezomib; OPM-2 and KMS-11 for Eer1). Once again, single or dual inhibition had no detectable effect on S6P in fibroblasts. These findings provide further evidence that considerable differences can exist between MMCs in the way they respond to inhibition of the proteasome or p97. They also indicate that separate inhibition of the proteasome or p97 often results in mild effects on protein translation control pathways in MMCs. Some of the changes, such as increases in eIF2α phosphorylation, are consistent with adaptive UPR signalling. In contrast, dual inhibition invariably causes substantial changes that are consistent with a terminal translational shut-down in these pathways in MMCs. This interpretation fits with the loss of ribosomes from the cytosolic surface of the ER, vacuolisation of the ER, and the reduction in Akt levels described above. In fibroblasts the effect of dual p97/protesome inhibition on these pathways was very moderate and indicative of an adaptive response.

In the light of the activation of caspase-3 that we observe and the previously described role of JNK in bortezomib-induced cell death [52], we next analysed JNK- and caspase-dependence of MMC death induced by dual ERAD inhibition. MMC lines were pre-incubated with the JNK inhibitor, SP600125, and the pan-caspase inhibitor, zVADfmk and then treated with Eer1 and bortezomib. We observed that apoptosis of MMC lines induced by dual p97/proteasome inhibition (Figure 3B) was JNK-independent (OPM-2 and KNS-11 cells) or partly JNK dependent (RPMI 8226 and U-266 cells), and caspase-dependent (RPMI 8226) or partly caspase-dependent (OPM-2, U-266, KMS-11).

### Structurally different inhibitors of p97 have comparable effects in MMC lines and primary MMCs

**Figure 4:**
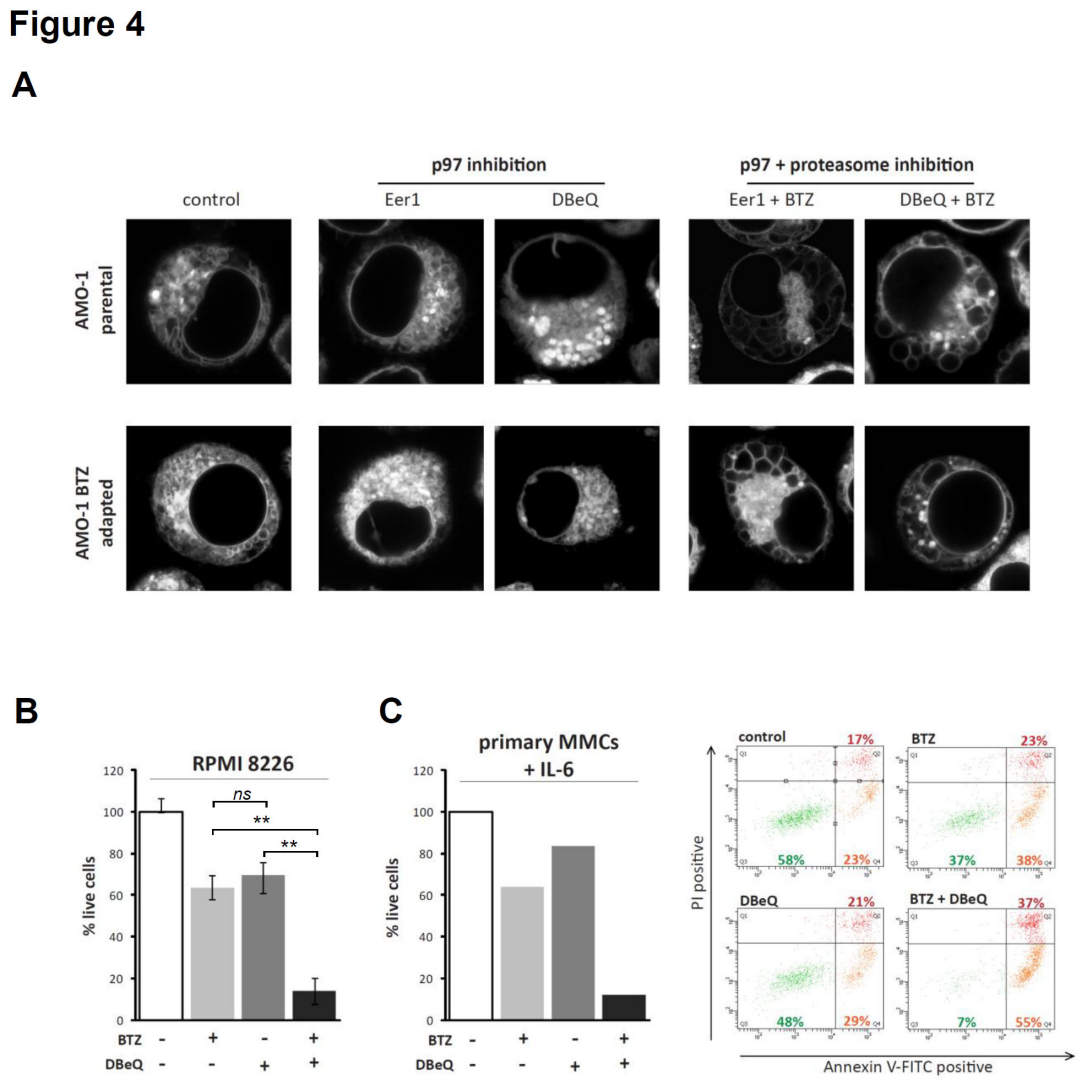
Structurally different ERAD inhibitors have comparable effects in MMCs. (**A**) Representative microscopic images of parental and BTZ-adapted AMO-1 cells stained with ER Tracker Blue-White DPX after treatment for 18h with Eer1 (5µM), DBeQ (5µM) or Eer1/DBeQ plus Bortezomib (5nM). The images show small to medium-sized globular/vesicular ER after p97 inhibition and ER vacuolisation after dual ERAD inhibition. (**B**) The proportion of live RPMI8226 cells was determined after treatment with bortezomib (10nM), DBeQ (10µM), or both, for 4h followed by drug wash-out and a further 20h incubation in culture medium (mean and SEM of 3 experiments; *p<.05, **p<.001, two-sided student’s t-test). (**C**) Primary MMCs were isolated from a bone marrow aspirate, incubated in culture medium containing IL-6 (10ng/ml) for 6h, followed by addition of bortezomib (5nM), DBeQ (5µM), or both for 24h and analysis of apoptosis levels. Live cells (Annexin V-FITC and PI-negative) are shown in green in the left lower quadrant, early apoptotic (Annexin V-FITC positive, right lower quadrant) and dead (Annexin V-FITC and PI-positive, right upper quadrant) cells are shown in orange and red, respectively.

Depletion of p97 by conditional gene knockout or RNAi knockdown has limited utility for predicting the effects of pharmacological p97 inhibition due to slow and incomplete impact on p97 function [27]. To investigate whether the effects of Eer1 described in the preceding sections are due to specific inhibition of p97, and to exclude potential off-target effects related to its chemical structure, we tested another recently identified p97 inhibitor, DBeQ [26,29]. Staining of parental and bortezomib-adapted AMO-1 MMCs with ER Tracker Blue-White DPX demonstrated that the effect of DBeQ on ER configuration was comparable to that of Eer1, with most of the ER presenting as small vesicular or globular structures (Figure 4A). Furthermore, combined treatment with both bortezomib and DBeQ resulted in vacuolar transformation of the ER that was very similar to the effect of combined Eer1 and bortezomib treatment (Figure 4A). Similar to Eer1, DBeQ substantially increased bortezomib-induced death of RPMI8226 cells and primary MMCs co-cultured with IL-6 (Figure 4B and 4C). The observation that the majority of the dying primary MMCs stained positive for Annexin V-FITC but excluded PI supports the notion that MMC death induced by dual ERAD inhibition is apoptotic. Thus, structurally different inhibitors of p97 have comparable effects on ER configuration and viability of MMCs, alone or in combination with bortezomib.

## Discussion

The key finding of this paper is that all of the MMCs that we have tested show dramatic changes to the ER in response to dual inhibition of p97 and the proteasome, and that this is accompanied by disruption of intracellular protein metabolism and cell death. Importantly, the toxic effects of dual p97 and proteasome inhibition are also observed for cell lines that are insensitive to bortezomib. The effects of dual inhibition are quite different from the effects of single inhibition of p97 or the proteasome, adding significant information on the non-redundant roles of p97 and the proteasome in maintaining the integrity of the secretory apparatus.

Dual p97/proteasome inhibition causes changes in key protein translation pathways in MMCs that are consistent with a terminal translational shut-down. This interpretation is based on the observation of proteolytic processing of eIF2α, and loss of both phosphorylated and total S6P and Akt [48-50]. It is also supported by the loss of ribosomes from the cytosolic surface of the ER observed by TEM. In contrast, inhibition of either the proteasome or p97 had a relatively modest impact on these protein translation control pathways and did not result in loss of ribosomes from the cytosolic surface of the ER. In particular, bortezomib or p97 inhibition alone did not cause proteolytic processing of eIF2α but moderately increased its phosphorylation on Ser51 in some MMC lines, indicating activation of adaptive PERK-dependent UPR signalling.

ER staining and electron microscopy showed that proteasome inhibition had a very mild impact on the appearance of the ER in MMCs, while p97 inhibitors caused clear alterations to ER structure. The effect of p97 inhibition on ER structure could reflect a hitherto unrecognised role for p97 in the metabolism of protein components of the ER membrane or of proteins that play a role in maintaining ER integrity. Such functions are suggested by the requirement of a p97/p37 complex for Golgi and ER maintenance in interphase [23]. The dramatic transformation of the ER into very large vesicles that we observed as a consequence of dual p97/proteasome inhibition was unexpected. It is possible that the disruption of ERAD increases drastically the amount of misfolded proteins inside the ER, causing an increase in osmolarity in the ER lumen that leads to an influx of water from the cytosol. Another explanation might be that structural ER proteins are affected by the disruption of protein translation. One mechanistic question that arises from our findings is whether the translational shut-down and the disruption of ER structure that result from dual p97/proteasome inhibition are responsible for triggering cell death or occur downstream from apoptotic signalling. The previously described cleavage of eIF2α by caspase-3 might seem to favour the latter scenario [48]. However, the pan-caspase inhibitor zVADfmk did not block the formation of large ER vacuoles after dual p97/proteasome inhibition. Furthermore, at the time-point when cleavage of eIF2α was evident in OPM-2 cells, the cells that presented with large ER vacuoles and had lost ribosomes from the ER surface did not yet show signs of apoptotic chromatin condensation. These findings suggest that the structural changes to the ER and the disruption of protein translation are not merely a consequence of apoptotic signalling. We therefore favour a model in which alterations of ER structure and protein translation play an important early role in triggering cell death, but are also aggravated further by the initiation of apoptotic signalling, culminating in irreparable disruption of protein translation and ER homeostasis.

Another important finding of this study is that the deleterious effects of dual inhibition of p97 and the proteasome occur preferentially in MMCs. In fibroblasts, dual p97/proteasome inhibition failed to induce activation of caspase-3 and did not affect levels of phosphorylated or overall Akt and S6P. Moreover, dual inhibition did not induce proteolytic processing of eIF2α in fibroblasts but increased its phosphorylation, indicating PERK-dependent adaptive UPR signalling. Furthermore, dual p97/proteasome inhibition was only moderately toxic to primary PBMNCs. Taken together, these findings indicate that dual p97/proteasome inhibition shows a considerable degree of selectivity towards MMCs.

It is also noteworthy that the toxic effects of dual p97/proteasome inhibition on MMCs were achieved at bortezomib concentrations similar to steady-state plasma concentrations maintained in patients after the early peak [53]. This is relevant in the light of evidence that peripheral neuropathy, a major side effect of bortezomib treatment in patients, may be caused by off-target effects at high peak plasma concentrations [53,54]. Additional *in vivo* analysis will be required to elucidate potential toxic and anti-myeloma effects of pharmacological p97 inhibition, alone and in combination with bortezomib. Systemic *in vivo* studies are currently not possible due to the limited solubility of Eer1 and DBeQ. However, the findings presented here form a conceptual preclinical framework for combined targeting of p97 and the proteasome as a potential novel therapeutic approach in MM and provide a strong incentive for developing p97 inhibitors that can be administered systemically [27].

In contrast to the clinical effectiveness of proteasome inhibitors in MM, they have shown disappointing results in most other haematological malignancies and solid cancers. The potent activity of p97 inhibitors in various cancer cells suggests that p97 might be a more broadly useful target for antineoplastic therapy than the proteasome [11,26,55]. One possible reason for this is that inhibition of p97 disrupts ERAD upstream of the proteasome, making its effects potentially independent from variations in proteasomal capacity, which can render cells insensitive to proteasome inhibition [5]. Inhibition of p97 may also be more effective than proteasome inhibition in disrupting protein homeostasis because it interferes with both HDAC6-mediated aggresome formation and autophagosome maturation, which are potential alternatives to proteasomal degradation of waste proteins [13-15,26,33,56]. In addition, p97 inhibition directly regulates intracellular levels of p53 and NFκB, which has been shown to mediate the toxic effects of Eer1 active against non-small cell lung carcinoma (NSCLC), in which p97 is overexpressed [55]. The exceptional secretory load of MMCs, combined with their high dependence on proteasomal and non-proteasomal protein degradation pathways, provides a plausible explanation for the dramatic effects of combined p97 and proteasome inhibition on the secretory apparatus that we report here. Considering that p97 has key roles in a diverse range of cellular processes, it will be important to investigate the combination of p97 inhibitors with other classes of anti-neoplastic drugs in different types of cancers [22,30,32,57].

## Supporting Information

Figure S1 **Effect of single and dual p97/proteasome inhibition on ER structure in MMC lines.** OPM-2, U-266, and KMS-11 cells were stained with ER Tracker Blue-White DPX following treatment for 24h (14h for KMS-11) with BTZ (5nM), Eer1 (5µM), or both. Representative confocal microscopic images are shown.
(TIF)

Figure S2 **ER vacuolisation caused by dual ERAD inhibition is not caused by overwhelming ER stress or apoptotic signalling.** OPM-2 cells were stained with ER Tracker Blue-White DPX following treatment for 24h with high doses of BTZ, Eer1, and tunicamycin, or BTZ+Eer1 after pretreatment with the pan-caspase inhibitor zVADfmk.
(TIF)

Figure S3 **Representative transmission electron miscrocopic images of OPM-2 cells treated with Bortezomib (5nM) and Eer1 (5µM) for 24h.**
(TIF)
