# Supplementary figures and images for: Combined Inhibition of p97 and the Proteasome Causes Lethal Disruption of the Secretory Apparatus in Multiple Myeloma Cells
Source: PLoS One. 2013 Sep 17;8(9):e74415. doi: 10.1371/journal.pone.0074415 (PMC3775786; doi:10.1371/journal.pone.0074415)

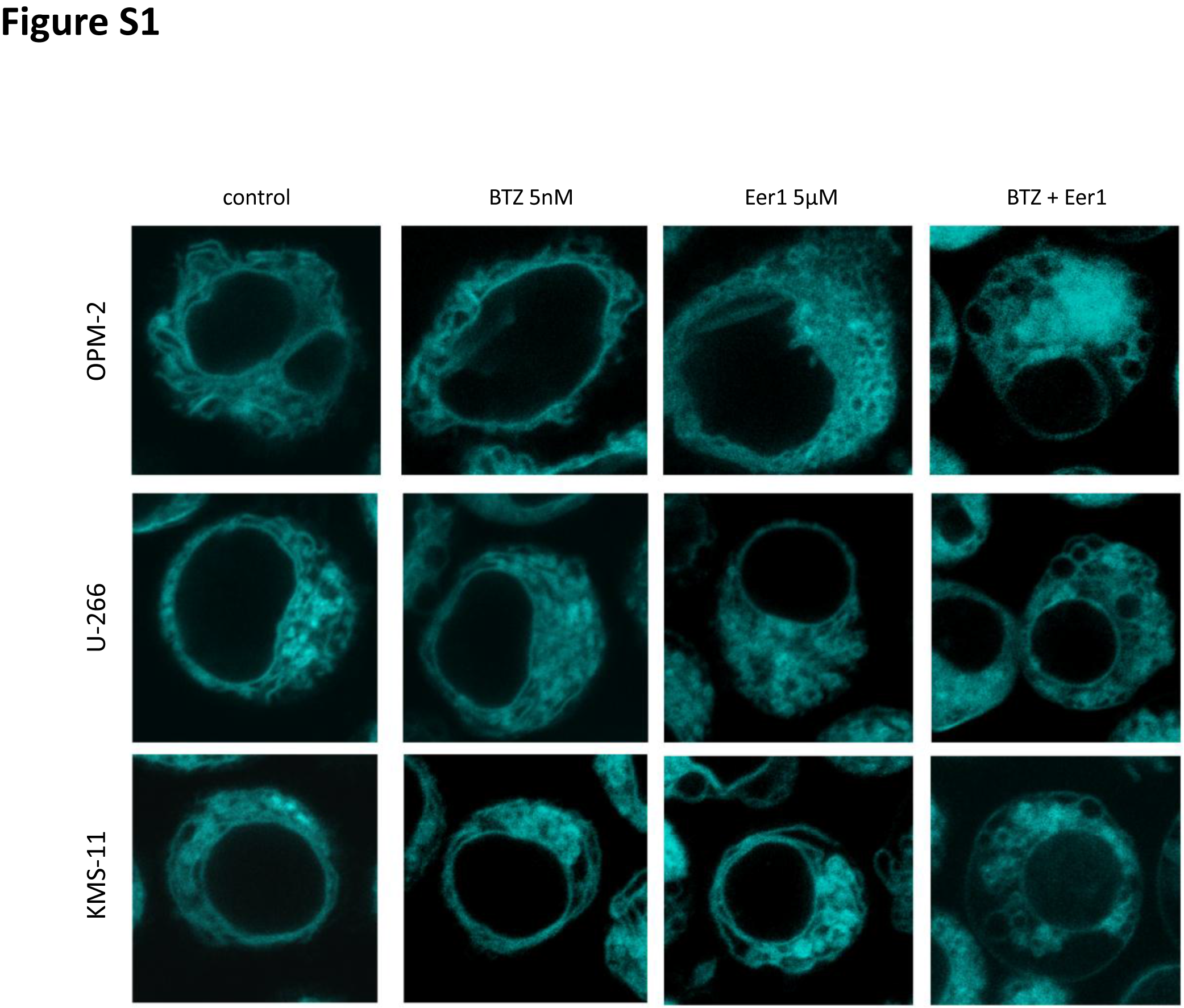

Supplement: Figure S1 — Effect of single and dual p97/proteasome inhibition on ER structure in MMC lines. OPM-2, U-266, and KMS-11 cells were stained with ER Tracker Blue-White DPX following treatment for 24h (14h for KMS-11) with BTZ (5nM), Eer1 (5µM), or both. Representative confocal microscopic images are shown. (TIF) [file pone.0074415.s001.tif]

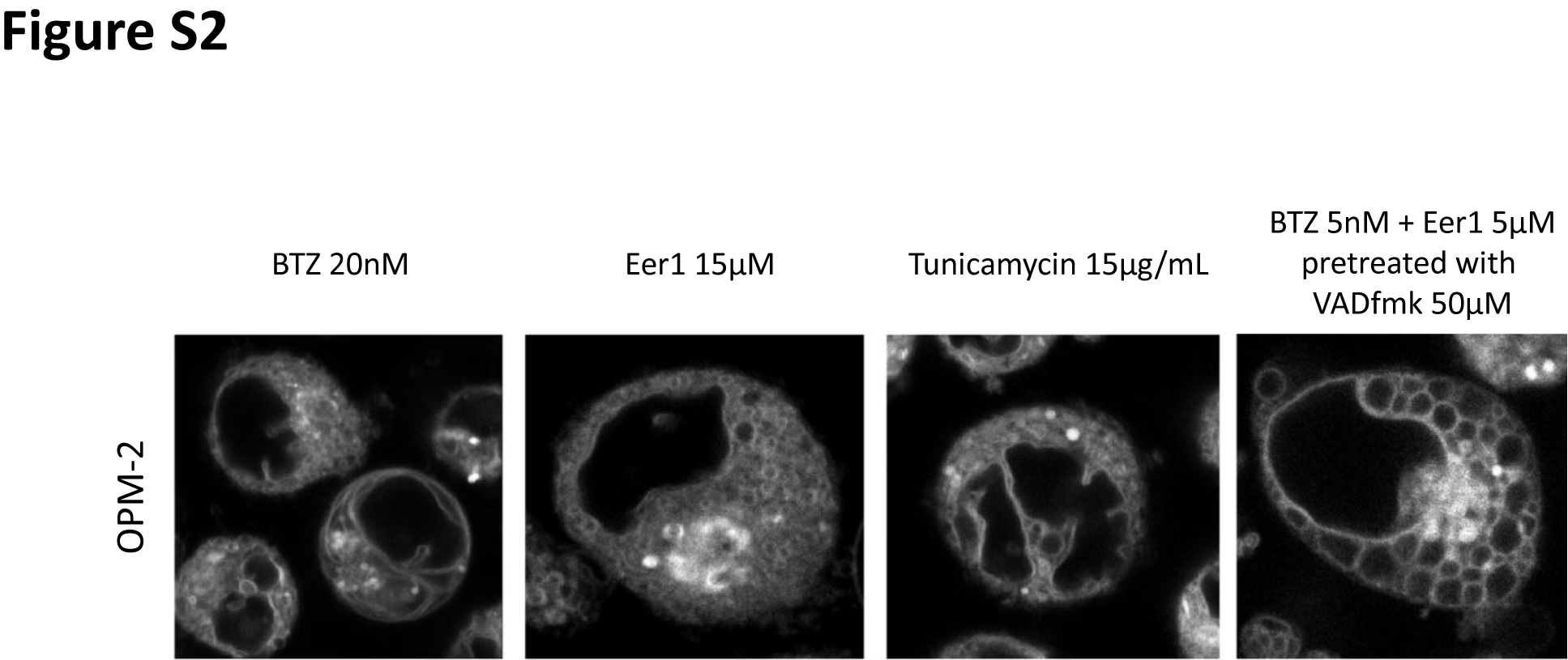

Supplement: Figure S2 — ER vacuolisation caused by dual ERAD inhibition is not caused by overwhelming ER stress or apoptotic signalling. OPM-2 cells were stained with ER Tracker Blue-White DPX following treatment for 24h with high doses of BTZ, Eer1, and tunicamycin, or BTZ+Eer1 after pretreatment with the pan-caspase inhibitor zVADfmk. (TIF) [file pone.0074415.s002.tif]

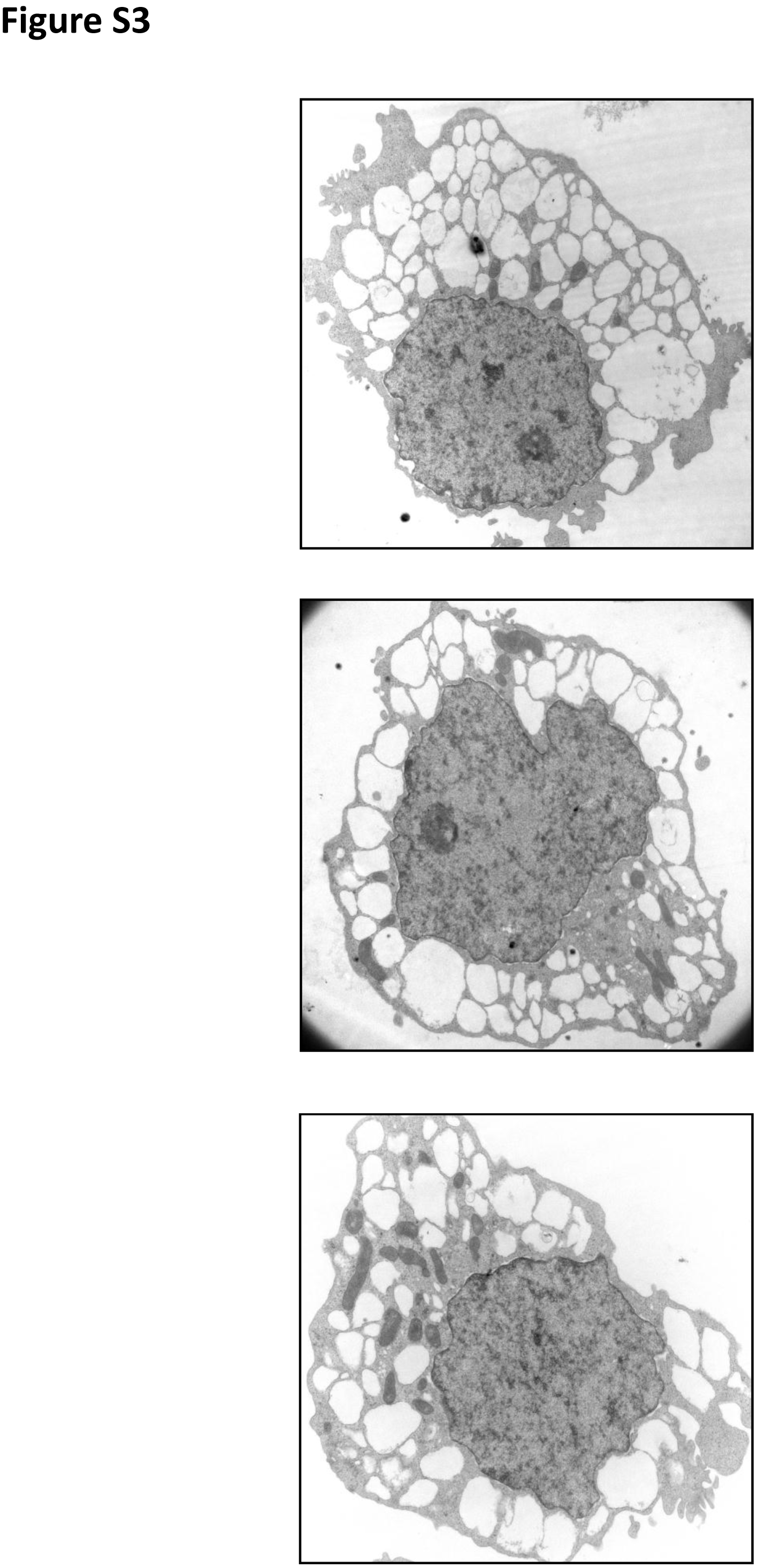

Supplement: Figure S3 — Representative transmission electron miscrocopic images of OPM-2 cells treated with Bortezomib (5nM) and Eer1 (5µM) for 24h. (TIF) [file pone.0074415.s003.tif]
